# A p53-like transcription factor, BbTFO1, contributes to virulence and oxidative and thermal stress tolerances in the insect pathogenic fungus, *Beauveria bassiana*

**DOI:** 10.1371/journal.pone.0249350

**Published:** 2021-03-31

**Authors:** Juan-Juan Wang, Ya-Ping Yin, Ji-Zheng Song, Shun-Juan Hu, Wen Cheng, Lei Qiu

**Affiliations:** 1 School of Biological Science and Technology, University of Jinan, Jinan, China; 2 State Key Laboratory of Biobased Material and Green Papermaking, Qilu University of Technology, Shandong Academy of Sciences, Jinan, China; 3 Maize Research Institute, Shandong Academy of Agricultural Sciences, Jinan, China; University of California Riverside, UNITED STATES

## Abstract

The p53-like transcription factor (TF) NDT80 plays a vital role in the regulation of pathogenic mechanisms and meiosis in certain fungi. However, the effects of NDT80 on entomopathogenic fungi are still unknown. In this paper, the NDT80 orthologue BbTFO1 was examined in *Beauveria bassiana*, a filamentous entomopathogenic fungus, to explore the role of an NDT80-like protein for fungal pest control potential. Disruption of *BbTFO1* resulted in impaired resistance to oxidative stress (OS) in a growth assay under OS and a 50% minimum inhibitory concentration experiment. Intriguingly, the oxidation resistance changes were accompanied by transcriptional repression of the two key antioxidant enzyme genes *cat2* and *cat5*. Δ*BbTFO1* also displayed defective conidial germination, virulence and heat resistance. The specific supplementation of *BbTFO1* reversed these phenotypic changes. As revealed by this work, BbTFO1 can affect the transcription of catalase genes and play vital roles in the maintenance of phenotypes associated with the biological control ability of *B*. *bassiana*.

## Introduction

The p53-like transcription factor (TF) superfamily includes the NDT80/PhoG-like DNA-binding family (http://pfam.xfam.org/family/PF05224), which has only been discovered in unikont lineages [1]. NDT80-like proteins have different functions in different fungi, such as regulation of sexual development, meiosis, filamentation, virulence, drug resistance, programmed cell death and the response to nutrient stress [1, 2]. The NDT80 protein of *Saccharomyces cerevisiae* is considered to be the founding member of the NDT80 family within the p53-like superfamily [1] and a key regulator of sporulation and meiosis [3]. XprG, also known as an NDT80-like protein, positively regulates mycotoxin generation, carbon depletion-induced apoptosis, and extracellular protease expression in *Aspergillus nidulans* [4]. This protein is similar to *Neurospora crassa* VIB-1, which plays a role in gene expression related to the positive regulation of extracellular protease generation as well as heterokaryon incompatibility-related apoptosis [2, 3, 5]. In *Candida albicans*, the roles of three NDT80-like proteins have been extensively studied. As regulators, CaNDT80 and CaRep1 control transporters (CDR1 and MDR1), which can eliminate drugs [6, 7]. Mutations in *CaNDT80* and *CaRON1* affect the induction of hyphal growth [7]. The *CaNDT80* mutant also shows weakened virulence in a mouse model of infection [8]. *NDT80* homologue mutations have certain impacts on secondary metabolism, including asexual or sexual spore pigmentation. *N*. *crassa* Δ*vib-1* mutants show pinkish conidia but not orange conidia (asexual spores); by contrast, mutants of XprG in *A*. *fumigatus* and *A*. *nidulans* possess pale conidia [3, 9]. Moreover, NDT80-like proteins are involved in fungal nutrition sensing. For example, suppressor of fusion (Suf), the Ndt80 homologue in *Fusarium oxysporum*, plays a vital role in regulating anastomosis depending on the nutrient [10]. As far as *Trichoderma reesei* is concerned, this protein also plays an important role in activating the *N*-acetylglucosamine (GlcNAc) gene cluster [2, 11]. *C*. *albicans* Rep1 and *A*. *nidulans* XprG are also required for GlcNAc catabolism [7].

The genus *Beauveria*, including the species *B*. *bassiana*, represents a strong insect pathogen with a wide range of hosts and has been prepared as a candidate mycoinsecticide with environmental friendliness [12]. As an important filamentous insect fungal pathogen, *B*. *bassiana* is the typical model for examining the pathogenic mechanism and growth of fungi together with their interactions with the host [13, 14]. Recently, some TFs in *B*. *bassiana* have been characterized. Typically, the *BbMbp1* gene facilitates the transition of *B*. *bassiana* morphology with regard to saprophytic and pathogenic growth through a variety of genetic pathways [15]. In addition, in *B*. *bassiana*, the MADS-box TF Bbmcm1 plays a vital role in the regulation of the cell cycle, virulence and integrity [16]. Moreover, *BbOhmm* affects the homeostasis of reactive oxygen species (ROS) combined with the available oxygen to control *BbSre1* transcriptional activity, thus regulating the homeostasis of mitochondrial iron, respiration-related genes and haem production to adapt to hypoxic conditions [14].

In our study, it is shown that the NDT80-like protein in *B*. *bassiana* plays an important role in oxidative stress (OS) resistance by impacting the expression of key catalase genes. Therefore, this protein was designated TFO1 (transcription factor regulating oxidation) in *B*. *bassiana*. This study was conducted to examine how BbTFO1 affects *B*. *bassiana* phenotypic changes related to its biological control ability through multi-phenotypic analyses of specific gene deletion or complementation strains combined with the parental WT. The results indicate that BbTFO1 plays an important role in regulating antioxidant effects, thermotolerance and pathogenesis in insect fungal pathogens.

## Materials and methods

### Phylogenetic analysis of BbTFO1

In NCBI databases, using the *S*. *cerevisiae NDT80* sequence as a query, BLAST analysis based on NCBI was carried out to identify the genome from ARSEF 2860, the wild-type *B*. *bassiana* strain (WT), and some typical filamentous fungi. Next, each NDT80 homologous protein sequence found in the fungi was aligned to compare the structures and analyse the phylogenetics using MEGA7 (http://www.megasoftware.net).

### Construction of *BbTFO1* deletion and complementation mutants

*BbTFO1* deletion together with a complementation mutant was created via *Agrobacterium*-mediated transformation [17]. The synthetic plasmids for homologous transformation were transformed by *Agrobacterium tumefaciens* and mobilized into *B*. *bassiana* according to a previous procedure [18]. Using the paired primers (Table 1), PCR was carried out to clone *BbTFO1* 5′ or 3′ coding/flanking fragment sequences based on the WT genome, with *TaKaRa La Taq*^®^ (TaKaRa, Japan) used as the catalyst, followed by specific enzyme digestion and insertion to the target plasmid to form target gene deletion plasmids. *BbTFO1* was knocked out in the WT by homogenously recombining the 5′ and 3′ fragments with a *bar* marker. The gene deletion mutant was initially screened by herbicide (200 μg ml^−1^ phosphinothricin) resistance [19].

**Table 1:** Paired primers used for gene cloning, deletion, complement and expression.

Primers	Paired sequences (5′-3′)*	Purpose
TFO1up-F/R	AAAGAATTCGAGCATCATCGCAGACTTG/AAAGGATCCGGATGGACAGGGAGGTAAA	Cloning 5′ *BbTFO1*
TFO1dn-F/R	AAATCTAGAGCGTGGTAACTCTGGAATG/AAAAGATCTGACGCTTGCTCGCTCTT	Cloning 3′ *BbTFO1*
TFO1fl-F/R	GGGGACAAGTTTGTACAAAAAAGCAGGCTGCACCAAAGGTTGAGATAGA/⋅ GGGGACCACTTTGTACAAGAAAGCTGGGTTCCAGAAACAGAACGAAAA	Cloning full-length *BbTFO1*
pTFO1-F/R	AAATCGTGCGGGTCGTGTTA/AAATGCTGTATTGGCGTTGC	PCR detecting *BbTFO1*
qTFO1-F/R	GAGGATTGCGGTGTCATA/CACTGCCACTATTCGGATA	qPCR detecting *BbTFO1*
q18S-F/R	TGGTTTCTAGGACCGCCGTAA/CCTTGGCAAATGCTTTCGC	qPCR detecting 18S rRNA
qcat1-F/R	CCGTCTGGGCATCAACTGGGAAG/GCTGGGCGTGGTCGTGGTAG	qPCR detecting *Bbcat1*
qcat2-F/R	CCTCTGACGTTGGCGGCCCTTTC/CCGTGTCCGTGCTGCCTCGTG	qPCR detecting *Bbcat2*
qcat3-F/R	GAGGAGCCCAGCAACGCACAAGAG/TGAGGACGACAAGGCCGCCATT	qPCR detecting *Bbcat3*
qcat4-F/R	CGGCTGCGGTGTCTTGTCCATAC/CCTTGTCGGCGTTCTGGCGAAG	qPCR detecting *Bbcat4*
qcat5-F/R	GCTGGGCTGATCTGCTGGTCCTTG/TCCTTGCTGTAACGGTGGCTGTCG	qPCR detecting *Bbcat5*
qcat6-F/R	TCAAGTCGGTTCAGGAGATGGAG/TTGTTGCGTCTTCAATCGGAGTG	qPCR detecting *Bbcat6*
qSod1-F/R	GCGGCTTCCACATCCACACCTTTG/GGTCCAGCGTTGCCAGTCTTGAG	qPCR detecting *BbSod1*
qSod2-F/R	CCAGTGTTTGGCATTGACATG/TCAGCCGTCTTCCAGTTGATG	qPCR detecting *BbSod2*
qSod3-F/R	TCTCCGGCAAGATTATGGAGC/TTGGCGTCATTCTTGGCCT	qPCR detecting *BbSod3*
qSod4-F/R	CGAGATGGTCCTTACGGCTTCAG/GCTCCCAGGTGTTGAGGCATAG	qPCR detecting *BbSod4*
qSod5-F/R	CGGCGACCTCAGCGGCAAGTAC/GCCAGCAACAACAGGGACCGTAGG	qPCR detecting *BbSod5*
qOhmm-F/R	CACGATATACAGAACCATTCC/CAGTCCATACACCACCTT	qPCR detecting *BbOhmm*

* Underlined regions suggest the sites of restriction enzyme in the *BbTFO1* deletion mutant (*Eco*RI/*Bam*HI and *Xba*I/*Bgl*II) or gateway fragments exchanged for the targeted *BbTFO1* complementation mutant.

For the reverse complement of the *BbTFO1* deletion mutants, using Gateway BP Clonase^™^ II Enzyme Mix (Shanghai, China), primers (Table 1) were used to clone the entire *BbTFO1* coding sequence based on the WT genome, followed by insertion into the p0380-sur-gateway for exchange for gateway fragments, and finally, p0380-sur-BbTFO1 was formed [20]. The plasmid was transformed in deletion via *Agrobacterium*-mediated transformation to form complementation strains of *BbTFO1*, and a putative complementary strain was selected by chlorimuron ethyl (10 μg ml^−1^) tolerance. Quantitative real-time PCR (qPCR) and PCR were used to detect the expected deletion and complementation strains.

### Multi-phenotypic assays

Phenotypic assays were performed to detect the potential heterogeneities in the phenotype of Δ*BbTFO1* compared with controls (WT and Δ*BbTFO1*/*BbTFO1*). All assays were repeated three times.

An appropriate amount of conidial suspension (1 μl, 10^7^ conidia ml^−1^, identical concentration hereafter) harvested from SDAY (4% glucose, 1% yeast extract, 1% peptone plus 1.5% agar) plates was spotted onto media containing CPZ (3% sucrose, 0.1% K_2_HPO_4_, 0.3% NaNO_3_, 0.05% MgSO_4_, 0.001% FeSO_4_, 0.05% KCl combined with 1.5% agar), SDAY and 1/4 SDAY (containing 1/4 of every SDAY nutrient). The diameter of each colony was determined, and photos were taken at day 7 after incubation at temperatures of 25, 30 and 35°C. Additionally, the cross-sectional size of the colony was determined to be the growth rate index of diverse plates.

For chemical stresses, an appropriate amount of conidium suspension (1 μl) was added to CPZ plates containing different chemical stresses, including oxidative (2 mM H_2_O_2_ along with 0.2 mM menadione), cell wall (30 μg ml^−1^ Congo red) and osmotic (0.5 M NaCl) stressors. The colony diameters were measured and photographed as described earlier, and a CPZ plate without any chemical stress was used as the control.

To better assess how OS affected deletion mutant growth, an appropriate amount of conidium suspension (1 μl) was added to CPZ media containing gradient menadione doses (5–50 μM) and H_2_O_2_ (1–4 mM). The diameters of all colonies under OS and the control were measured to calculate the colony area after 7 days of incubation at 25°C. A minimum inhibitory concentration (MIC_50_) value of H_2_O_2_ and menadione that suppressed 50% colony growth was adopted as the OS resistance index in every strain.

Several conidial properties related to biocontrol potential were detected. The conidiation on SDAY was measured according to a related description [19]. Briefly, an appropriate amount of conidial suspension (100 μl) was uniformly added onto SDAY media, followed by incubation at 25°C. Then, three rounded cultures were collected from every plate every day by a cork borer (6 mm diameter) from day 5 onwards. The conidia on rounded cultures were completely added to 0.02% Tween-80 (1 ml). Later, conidial suspension content was assessed, followed by conversion to conidia count in the plate culture per unit area. In germination broth (GB: 0.5% peptone and 2% sucrose in 0.02% Tween-80), the median germination time (GT_50_, h) necessary to achieve 50% conidial germination under shaking at 110 rpm and 25/30°C was evaluated to be the conidial viability index at different temperatures [21].

The yield of blastospores was quantified in nitrogen-limited broth (NLB: 0.4% NH_4_NO_3,_ 4% glucose, 0.3% MgSO_4_, 0.3% KH_2_PO_4_). Briefly, 40 μl conidial suspensions were added to 20 ml of NLB and cultured at 25°C with shaking (110 rpm). The number of blastospores was counted after 3 days, and then the blastospore production was transformed into cell count per ml culture medium.

Conidial thermotolerance was evaluated using a previously described method [22]. Conidia tolerance to high temperature was defined as the median lethal time (LT_50_, min) when conidia were treated with 0–120 min heat stress exposure at 45°C. In this process, 100 μl samples were taken from each tube at 15 min intervals, and then 1 ml of GB was added. Afterwards, the sample was subjected to 24 h of shaking at 110 rpm and 25°C, and a cytometer was used to evaluate the conidial germination rate microscopically.

### Fungal virulence

*Galleria mellonella* larvae were used to assay fungal virulence according to immersion and injection approaches. The larvae were soaked in conidia suspension for 10 seconds and dry paper towels were used to remove excess liquid from the larvae. For intrahaemocoel injection, 1 μl conidial suspension (5 × 10^5^ conidia ml^−1^) was added to the larval haemocoel. The two control groups were exposed to sterile 0.02% Tween-80. Each experiment was carried out three times, with 30 larvae being adopted in every experiment. Moreover, the number of dead larvae was measured at intervals of half a day. After analysing the time-mortality trend, the LT_50_ (d) for every strain was determined against every larval group. After curing under the optimal conditions for 6 days, the fungal growth on the surface of the mummified cadavers was photographed. During fungal virulence assessment, haemolymph samples were extracted from larvae that survived 72 h after injection, and hyphal bodies were formed *in vivo*, as seen by laser scanning confocal microscopy (LEICA DMi8, Germany).

### Transcript profile analysis of antioxidant-related genes

The CAT family, SOD family and *Bbohmm* involved in OS responses were analysed by qPCR. In brief, 100 μl of conidial suspensions were inoculated into CPZ medium containing H_2_O_2_ (2 mM). After growing at 25°C for 5 days, an RNAiso Plus Kit (TaKaRa, Dalian, China) was utilized to extract total RNA from Δ*BbTFO1* and the WT strain. Then, a PrimeScript^RT^ reagent kit (TaKaRa) was utilized for cDNA synthesis by reverse transcription. The resultant cDNA was then diluted at 10 ng ml^-1^ and adopted as a template in qPCR by the use of paired primers (Table 1) [23]. For *B*. *bassiana*, 18S rRNA was applied as the internal standard of each transcript. The gene transcriptional level was determined and repeated three times. The relative transcription level of the antioxidant-related genes was calculated as the fold change of the mutant strain transcript relative to the WT strain transcript in stress culture.

### Statistical methods

One-way ANOVA, as well as Tukey’s HSD test, were used to analyse phenotype heterogeneities in the WT compared with control strains.

## Results

### BbTFO1 characteristics in and generation of *BbTFO1* mutants

Using the NDT80 sequence of *S*. *cerevisiae* as a query, the annotated *B*. *bassiana* ARSEF 2860 strain genome was searched against the NCBI database, generating the most highly related sequence of 502 amino acids (identity: 31%; E-value: 3e-05). Phylogenetic analysis (S1A Fig) showed that the BbTFO1 sequence had the closest relationship with NDT80 from *N*. *crassa* (XP 011394327) and *T*. *reesei* (XP 006961996) because they were located on the same branch. Similar to the transcription factor NDT80 of *S*. *cerevisiae*, BbTFO1 had an NDT80/PhoG-like DNA-binding domain (residues 131–318). Successful *BbTFO1* deletion and complementation were verified through PCR and qPCR using paired primers to examine correct recombination events (S1B–S1D Fig). Additionally, *BbTFO1* transcription was not detected in the deletion strains, but similar transcription levels were detected in the WT and complementary strains (S1D Fig).

### Contribution of BbTFO1 to chemical stress tolerance

**Fig 1:**
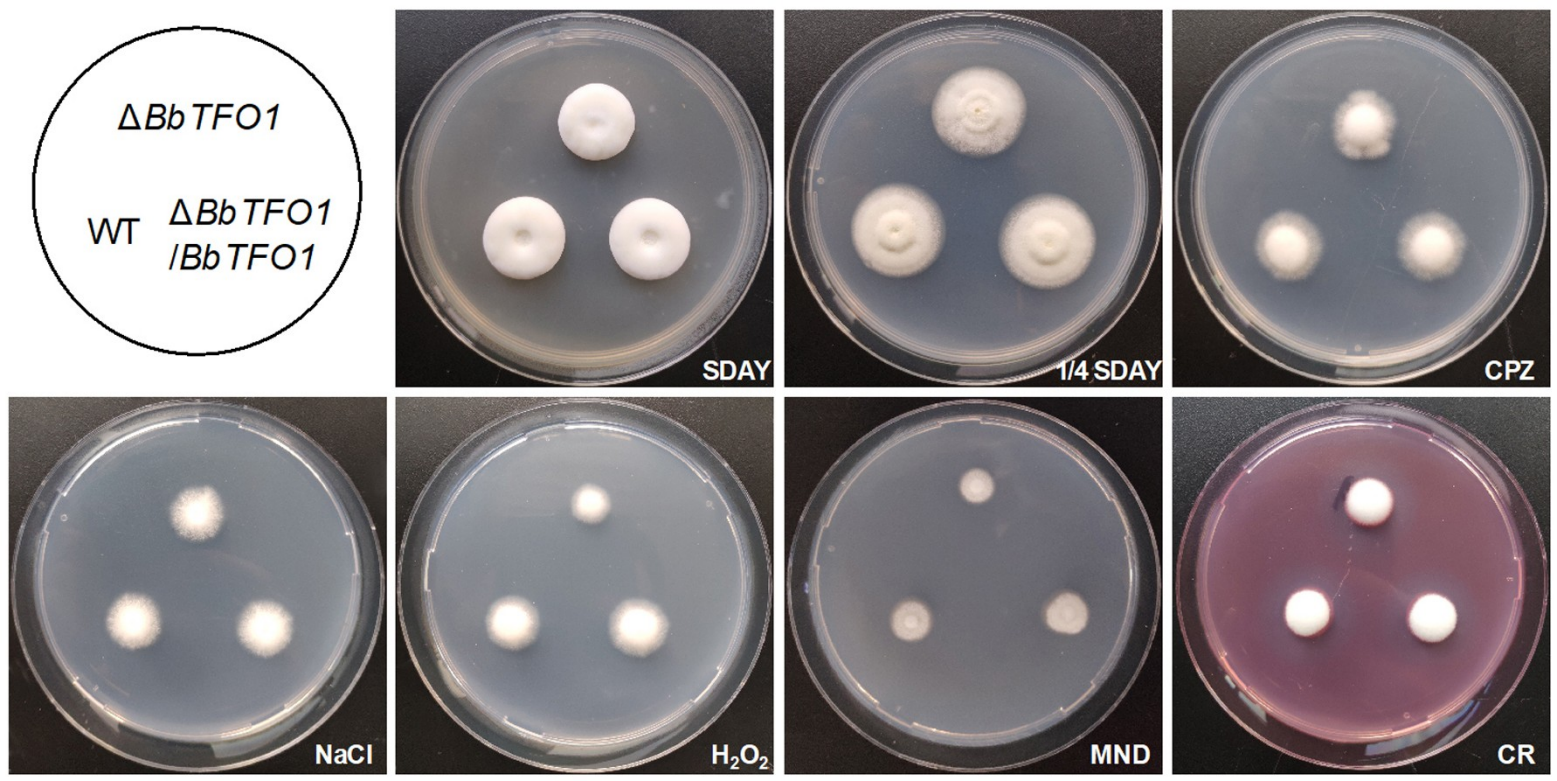
The growth of fungal colonies on SDAY, 1/4 SDAY, CPZ and CPZ containing NaCl (0.5 M), H_2_O_2_ (2 mM), menadione (MND: 0.2 mM) and Congo red (CR: 3 μg ml^−1^). The media were incubated for a period of 7 days at 25°C, and then photos were taken. Note that the Δ*BbTFO1* strains of *BbTFO1* grew smaller than the WT and Δ*BbTFO1*/*BbTFO1* strains on CPZ containing H_2_O_2_ and menadione.

The growth of deletion, complementation, and WT strains in different nutrient and chemical stress media is shown in Fig 1. The colony size of deletion strains was similar to that of control strains, and there was no significant difference with those in the rich medium SDAY, nutrient-deficient medium 1/4 SDAY, or minimal medium CPZ (Tukey’s HSD, *P* > 0.05) (Fig 1). Similar results were also found in CPZ medium containing NaCl and Congo red (Tukey’s HSD, *P* > 0.05). Nevertheless, Δ*BbTFO1* showed markedly higher sensitivity to OS than the control strains (Tukey’s HSD, *P* < 0.05). The above findings indicate that BbTFO1 plays an essential role in maintaining OS tolerance but not in responding to other nutritional and chemical stresses.

### Transcriptional changes in antioxidant-related genes in Δ*BbTFO1*

**Fig 2:**
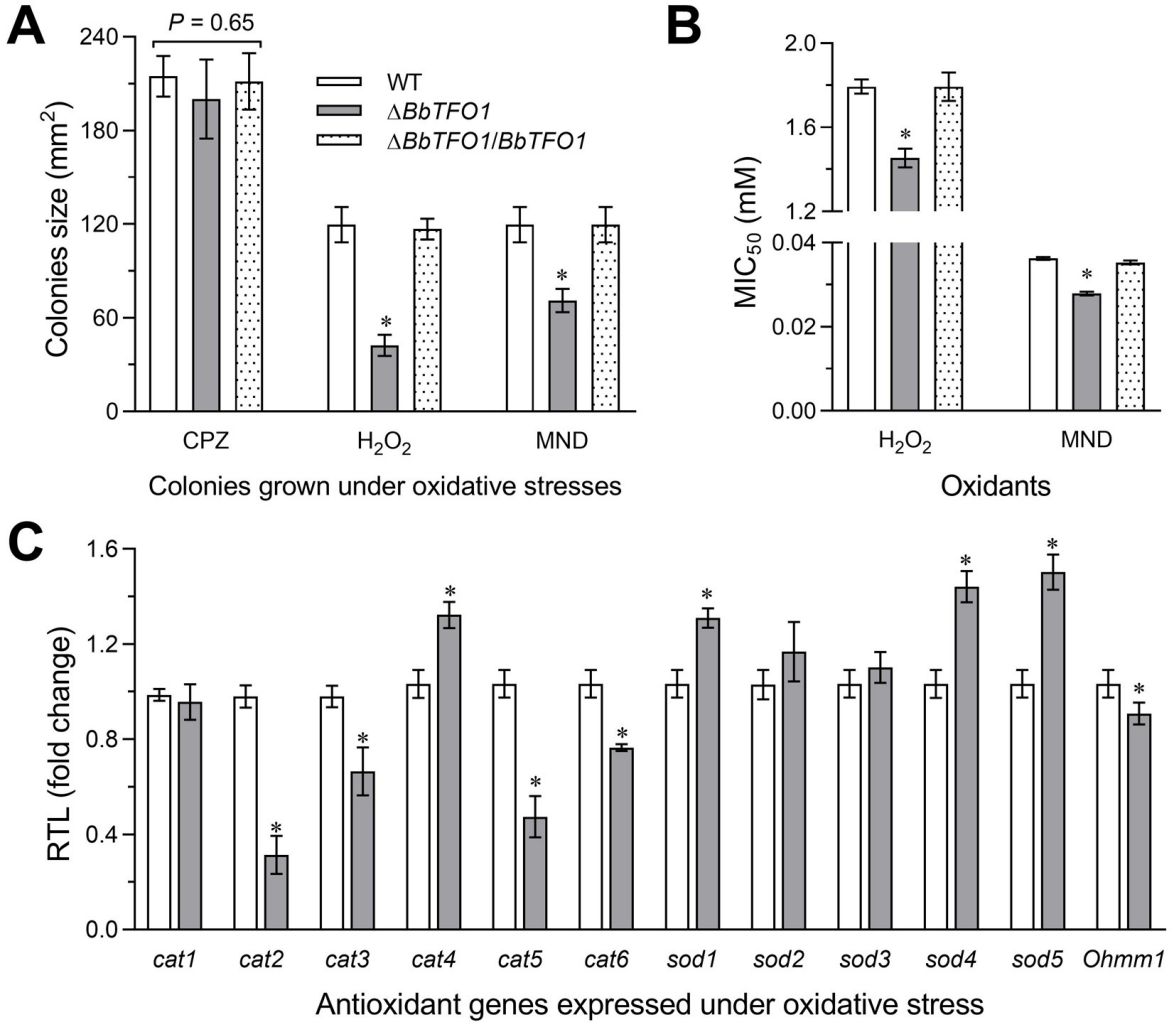
Disruptions to *BbTFO1* affect OS resistance and antioxidant-related enzyme gene expression in *B*. *bassiana*. (A) Colony size was measured on the seventh day of culture at 25°C after inoculation. (B) MIC_50_ estimates for two oxidants (H_2_O_2_ and menadione) to inhibit 50% fungal growth in diverse strains on CPZ at 25°C. (C) Relative antioxidant-related gene transcript levels of Δ*BbTFO1* strains compared with WT cultured for 5 days at 25°C on CPZ containing H_2_O_2_ (2 mM). The bar marked with an asterisk in each group shows a significant difference (Tukey’s HSD, *P* <0.05). Error bars: SD of 3 repeated assays.

The colony size of deletion strains decreased by 41% and 63% compared with the control in CPZ medium containing menadione and H_2_O_2_, respectively (Fig 2A). However, the colony size of the deletion strain was not significantly different from that of the control in CPZ medium (Fig 2A). Δ*BbTFO1* showed markedly high sensitivity to OS compared with controls. According to the MIC_50_ (Fig 2B), the deletion mutant also displayed high sensitivity to the oxidants H_2_O_2_ (19%) and menadione (23%). To explain the reduced tolerance of Δ*BbTFO1* to oxidative stress, the transcriptional levels of some antioxidant-related genes were analysed by qPCR. Among them, four of six CAT family genes and *Bbohmm* showed transcriptional downregulation compared with WT. Significantly, two key catalases, *cat2* and *cat5*, were transcriptionally repressed by 68% and 54%, respectively (Fig 2C), in response to OS in Δ*BbTFO1* [23]. However, three of five SOD family genes showed transcriptional upregulation compared with WT. These results suggest that Δ*BbTFO1* sensitivity to OS is accompanied by the transcriptional downregulation of these two key catalases.

### Role of BbTFO1 in conidiation and conidial quality

**Fig 3:**
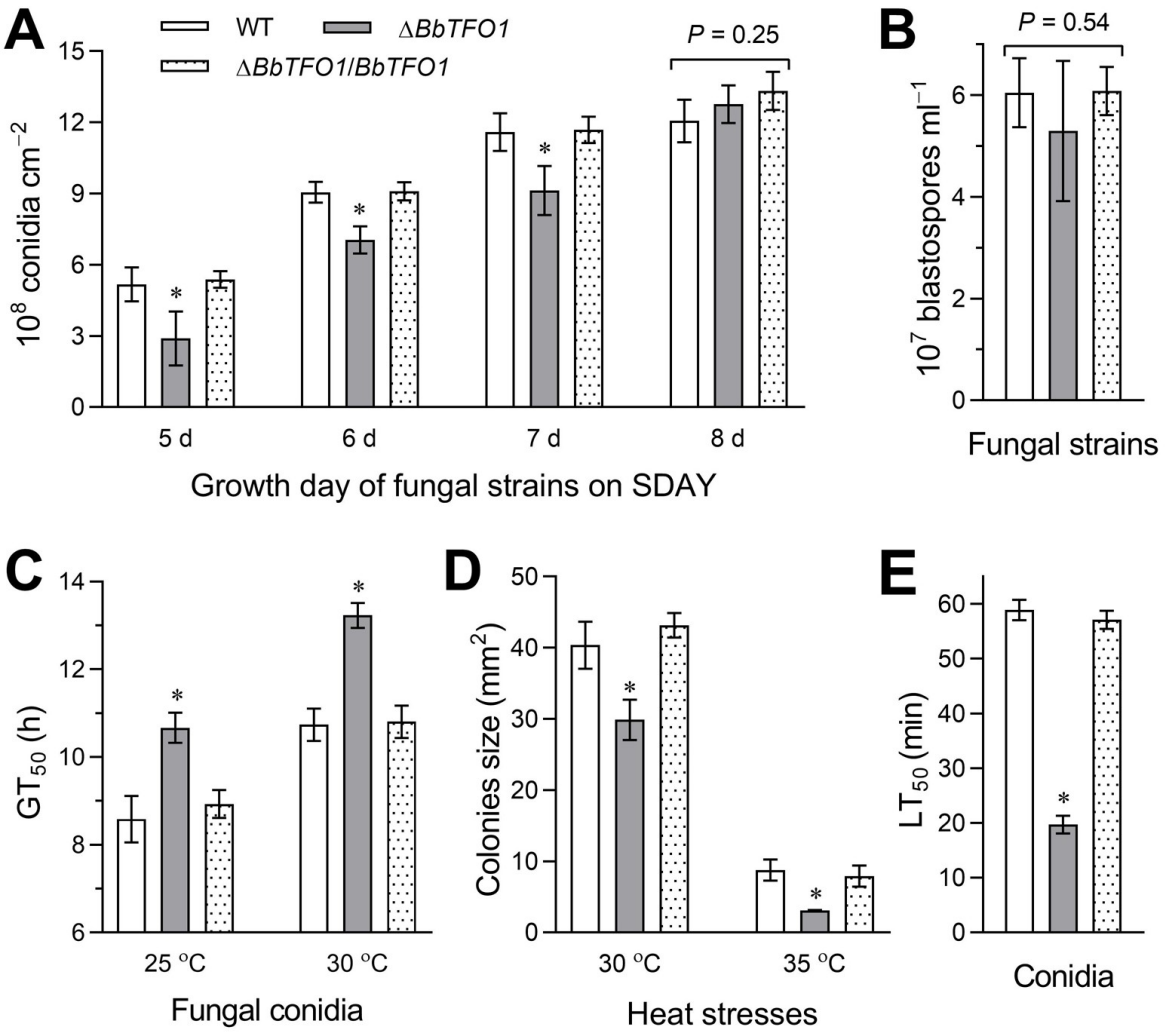
*BbTFO1* disruption affects fungal conidiation and conidial quality (A) Conidiation was counted daily from 5 days to 8 days on SDAY plates, which was started with smearing an appropriate amount of conidial suspension (100 μl, 10^7^ conidia ml^−1^). (B) The yield of blastospores was counted in NLB after 3 days of incubation at 25°C. (C) The GT_50_ (h) necessary to achieve 50% conidial germination at 25°C and 35°C. (D) Colony size in different strains grown at 30°C and 35°C for 7 days on CPZ medium. (E) The LT_50_ (min) of heat stress resistance in conidia at 45°C. The bar marked with an asterisk for every three-bar group showed a significant difference (Tukey’s HSD, *P* < 0.05). Error bars: SD of 3 replicates.

For conidiation, the Δ*BbTFO1* strain was decreased by 44%, 22% and 21% on days 5, 6, and 7 (Fig 3A), respectively, which was significantly different from the controls (Tukey’s HSD, *P* < 0.05). However, final Δ*BbTFO1* conidiation was similar to that of the control strain (Tukey’s HSD, *P* > 0.05). These results showed that the deletion of *BbTFO1* affected conidiation growth but not conidiation ability. Moreover, the production of blastospores at 25°C showed no significant difference in Δ*BbTFO1* compared with the corresponding controls (Tukey’s HSD, *P* > 0.05, Fig 3B).

Despite having no impact on conidiation capacity and the production of blastospores, deletion of *BbTFO1* exerts a marked influence on conidial germination and thermotolerance. The Δ*BbTFO1* mutant showed GT_50_ values of 2.1 h and 2.5 h longer than those of the WT at 25°C and 35°C, respectively (Fig 3C). The colony sizes of Δ*BbTFO1* decreased by 26% and 65% compared with WT at 30°C and 35°C, respectively (Fig 3D). Moreover, heat stress resistance of Δ*BbTFO1* at 45°C decreased significantly compared with that of the WT strain, and its LT_50_ decreased by 67% (Fig 3E). These data revealed the role of *BbTFO1* in conidial germination and its contribution to thermotolerance but not in the production of blastospores.

### Contribution of BbTFO1 to insect virulence

**Fig 4:**
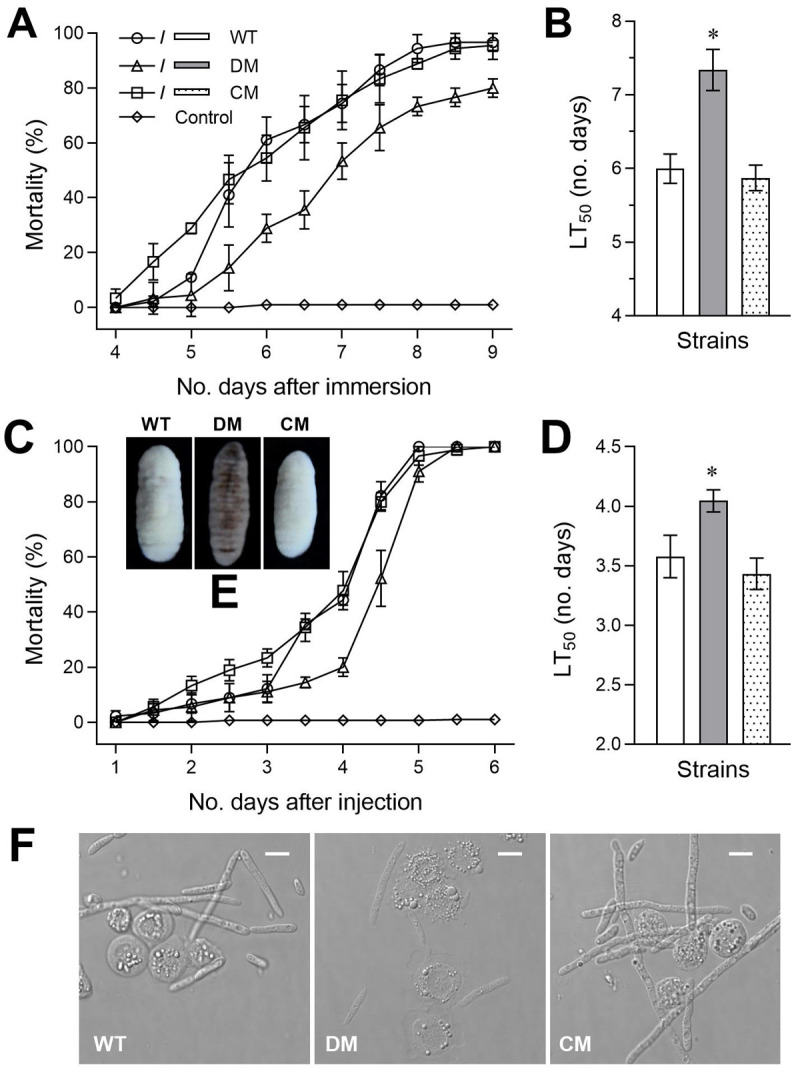
*BbTFO1* deletion leads to defects in fungal pathogenicity. (A, C) *G*. *mellonella* larval survival trend and (B, D) LT_50_ values after immersion in the 10^7^ conidia ml^−1^ suspension to normally infect the cuticle and injection with ~500 conidia/larva through the haemocoel to achieve cuticle-bypassing infection. (E) Fungal outgrowth images of Δ*BbTFO1* (DM), Δ*BbTFO1*/*BbTFO1* (CM) and WT on the cadaver surface at 6 days after death. (F) Microscopic images (scale: 10 μm) of hyphal bodies within haemolymph samples collected from the surviving larvae at 72 h after injection. Filamentous cells were hyphal bodies, while subspherical and spherical cells were host haemocytes. The bar marked with an asterisk of the three-bar group showed a significant difference (Tukey’s HSD, *P* < 0.05). Error bars: SD of 3 replicates.

For normal infection via the cuticular penetration method, time-mortality trends and means (± SD) LT_50_ of *G*. *mellonella* larvae following conidial suspension immersion are shown in Fig 4A and 4B. On the ninth day after immersion, the mortality of the WT and complementation strain was close to 100%, while that of Δ*BbTFO1* was approximately 80% (Fig 4A). The mean (± SD) LT_50_ values of the WT and complementation strains against larvae were 6.0 ± 0.2 and 5.9 ± 0.2 days via normal cuticle penetration (Fig 4B), respectively. The mean (± SD) LT_50_ values of the Δ*BbTFO1* mutant against larvae were extended until 7.3 ± 0.3 days (Fig 4B). For intrahemocoel injection, the mortality of the WT and complementation strains were close to 100% on the fifth day after injection (Fig 4C); the mean (± SD) LT_50_ values of the WT and complementation were 3.6 ± 0.2 and 3.4 ± 0.1 days, respectively; and the mean LT_50_ of Δ*BbTFO1* strains against the larvae increased to 4.0 ± 0.1 days (Fig 4D). After 6 days of preservation under the optimal conditions, the fungal growth on larvae infected with the control strain was greater than that on the larvae killed by Δ*BbTFO1* (Fig 4E).

To probe the potential reason for the reduced virulence by cuticle-bypassing infection with Δ*BbTFO1*, haemolymph samples obtained from larvae that survived 72 h after injection were analysed. As a result, there were rich hyphal bodies in the control strains but rare hyphal bodies within the haemolymph samples from the larvae following injection with Δ*BbTFO1* conidia (Fig 4F).

## Discussion

Compared with the controls, the Δ*BbTFO1* strains showed significant defects in tolerance to OS, together with certain typical defects in phenotype related to the biological control potential of fungi, such as virulence and conidial quality. Typically, two catalase genes were markedly downregulated in the deletion mutants. The complicated phenotypic changes caused by the deletion of *BbTFO1* are discussed below.

First, *B*. *bassiana* was subjected to biotic and abiotic OS in the field [24], which negatively affects the insect host infection capacity of fungi [25, 26]. Recently, more than 24 antioxidant enzymes showing dispersion within diverse families have been analysed for *B*. *bassiana* [27]. Some SODs in entomopathogenic fungi have been demonstrated to be crucial factors of fungal biocontrol potential [28]. Five members of the SOD family characterized in *B*. *bassiana* showed that Sod2 and Sod3 played a dominant role in fungal SOD activity [28, 29]. Each type of peroxide can be decomposed by members of the peroxidase (POD) family, including larger hydrogen peroxide, H_2_O_2_ and xenobiotics. KatG1, a bifunctional catalase-peroxidase, has been suggested to display POD and CAT activities, which play a vital role in chemical OS tolerance in *Metarhizium acridum* [24]. Both the GRX-GLR and TRX-TRR families have been examined to analyse their impacts on the antioxidation of *B*. *bassiana* [30, 31]. Some strong antioxidant genes have been adopted for entomopathogenic fungal genetic engineering for the sake of enhancing biological control abilities [32]. Overexpression of cytoplasmic manganese core superoxide dismutase (BbSod2) in *B bassiana* significantly increased antioxidant capacity, UV-B virulence and tolerance [33].

In addition, catalase family members decompose H_2_O_2_. Knockout mutants of catalases have shown discrete phenotypes in susceptibility to oxidative, heat, and UV-B stress in *B*. *bassiana* [34]. Among them, *cat5*, *cat1* and *cat4* are influential virulence factors, while *cat1* and *cat4* show equal importance to the regulation of UV-B resistance of conidia, with *cat2* ranking second. In addition, *cat1* is the most important regulator of conidial heat tolerance. It is believed that the CAT family affects fungal virulence by changing other virulence-related phenotypes of *B*. *bassiana* [23, 27]. Prior studies have shown that total catalase activity decreased by 89% and 56% in Δ*cat2* and Δ*cat5*, respectively, while this decrease was only 9–12% in additional *B*. *bassiana* catalase knockout mutants [23]. *cat2* and *cat5* exert viral parts in *B*. *bassiana* antioxidation of catalase family [23]. Moreover, *BbTFO1* deletion led to four of six CAT family genes showing transcriptional downregulation compared with the WT. In particular, the two key catalases *cat2* and *cat5* were transcriptionally repressed by 68% and 54%, respectively. In Δ*BbTFO1*, the defect of colony growth under OS may be due to the repression of catalase gene expression. Therefore, it was speculated that *BbTFO1* can contribute to fungal antioxidation by affecting the expression of catalase genes. Beyond expectation, the absence of *BbTFO1* did not increase the sensitivity to cell wall stressors in *B*. *bassiana*, while for additional fungi, such as *C*. *albicans*, *NDT80* deletion resulted in enhanced sensitivity to Congo red and SDS [2]. The lack of *NDT80* resulted in a denser biofilm relative to WT and might initiate the *TEC1*-dependent compensatory response, mainly through the *TEC1*-*ROB1* pathway [35].

Second, according to a previous report in *C*. *albicans* [2], *NDT80* deletion mutants also showed sensitivity to heat stress in *B*. *bassiana*. Heat stress could promote cellular ROS generation, thereby damaging certain biomolecules, such as DNA, lipids or proteins [33, 36]. Antioxidant enzymes can scavenge diverse intracellular ROS [27, 37]. Typically, SOD can decompose superoxide anions and produce H_2_O_2_ and oxygen [38], while H_2_O_2_ can be additionally decomposed to oxygen and water via catalase [39]. In the present work, 4 catalase gene expression levels, especially *cat2* and *cat5*, in Δ*BbTFO1* were repressed, resulting in a decrease in fungal ability to decompose H_2_O_2_, which naturally caused the reduced tolerance of Δ*BbTFO1* conidia to high temperature. In addition, *BbTFO1* knockout mutants were demonstrated to suffer defects in the development of conidiation and conidial germination. Similarly, the XprG mutant was slightly slower to initiate conidiation development in *A*. *nidulans* [4]. Conidial quality is also considered an essential virulence factor of *B*. *bassiana* [40]. In this study, defects in conidial quality may be one of the reasons for the decrease in virulence.

Finally, *BbTFO1* mutants showed reduced virulence to hosts in *B*. *bassiana* by immersion and injection, as observed in *NDT80* mutants of *C*. *albicans*. The virulence loss of *NDT80* mutants in *C*. *albicans* may be associated with the critical part of NDT80 in the control of hyphal growth and stress resistance [2, 8]. An NDT80 homologue in *F*. *oxysporum* (Suf, suppressor of fusion) is associated with horizontal transport of virulence gene-carrying small chromosomes under conditions with limited nutrients [10]. For *B*. *bassiana*, the host immune response will produce cytotoxic molecules such as ROS when it infects insects [41]. Host phagocytes also generate reactive oxygen intermediates (ROIs), which are toxic to a variety of microorganisms [42]. Based on this experiment, it was believed that the disruption of *BbTFO1* leads to a reduction in the transcription levels of four catalases and complex phenotypic changes, which are responsible for the reduction in fungal virulence. Catalase is considered a potential virulence factor in pathogenic fungi [23]. Overexpression of one catalase led to increased virulence of *B*. *bassiana* [43]. Catalase deletion within *A*. *fumigatus* mycelium and conidia leads to a significant decrease in virulence to mice [44]. In *Magnaporthe grisea*, the absence of *cat2* enhances fungal sensitivity to OS and significantly decreases virulence to barley [45]. An NDT80-like protein was further examined in *A*. *fumigatus*, a pathogenic fungus in animals, showing that XprG makes no difference in the virulence of mice [9]. The deletion of *BbTFO1* leads to defects in antioxidant capacity and germination defects, indicating that *BbTFO1* is a critical virulence factor of *B*. *bassiana*.

## Conclusion

Our results confirmed the previously reported functional diversity of NDT80-like proteins in different fungi [7]. In addition, BbTFO1 plays a crucial role in antioxidant activity, thermotolerance, conidiation, conidial quality and virulence in *B*. *bassiana*. According to transcript analysis, BbTFO1 can positively affect the expression of two key catalases to influence the antioxidant capacity of fungi, following the impact of the *B*. *bassiana* biological control potential.

## Supporting information

S1 Fig Bioinformatics description of BbTFO1 and generation of its mutant strains. (A) Phylogenetic tree of *B*. *bassiana* NDT80 homologues and additional fungi. The NCBI accession codes for all proteins, together with the sequence identities in *B*. *bassiana*, are provided in brackets after the fungal names. (B) Sketch map of *BbTFO1* deletion strategy. (C) *BbTFO1* deletion identification (lanes 1–3) by PCR. Lane M: DNA marker (bp). Lane CK: blank control. Lane 1: WT. Lane 2: Δ*BbTFO1*. Lane 3: Δ*BbTFO1*/*BbTFO1*. (D) Identification of *BbTFO1* deletion by qPCR.
(TIF)

S1 Raw images (PDF)
