# Supplementary figures and images for: A p53-like transcription factor, BbTFO1, contributes to virulence and oxidative and thermal stress tolerances in the insect pathogenic fungus, Beauveria bassiana
Source: PLoS One. 2021 Mar 31;16(3):e0249350. doi: 10.1371/journal.pone.0249350 (PMC8011754; doi:10.1371/journal.pone.0249350)

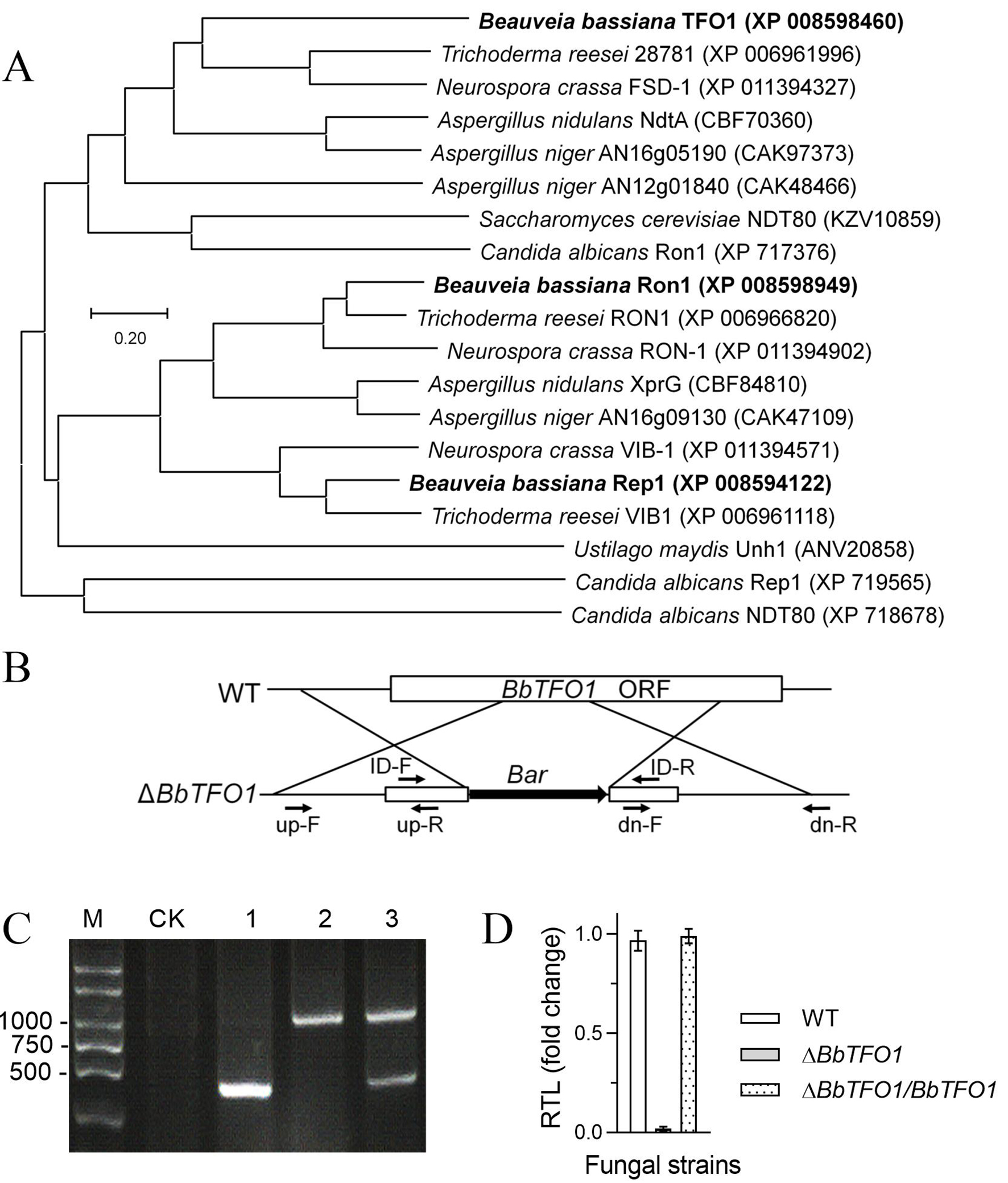

Supplement: S1 Fig — (A) Phylogenetic tree of B. bassiana NDT80 homologues and additional fungi. The NCBI accession codes for all proteins, together with the sequence identities in B. bassiana, are provided in brackets after the fungal names. (B) Sketch map of BbTFO1 deletion strategy. (C) BbTFO1 deletion identification (lanes 1–3) by PCR. Lane M: DNA marker (bp). Lane CK: blank control. Lane 1: WT. Lane 2: ΔBbTFO1. Lane 3: ΔBbTFO1/BbTFO1. (D) Identification of BbTFO1 deletion by qPCR. (TIF) [file pone.0249350.s001.tif]
