# Supplementary material for: A p53-like transcription factor, BbTFO1, contributes to virulence and oxidative and thermal stress tolerances in the insect pathogenic fungus, Beauveria bassiana
Source: PLoS One. 2021 Mar 31;16(3):e0249350. doi: 10.1371/journal.pone.0249350 (PMC8011754; doi:10.1371/journal.pone.0249350)
Supplement: S1 Raw images — (PDF) [file pone.0249350.s002.pdf]

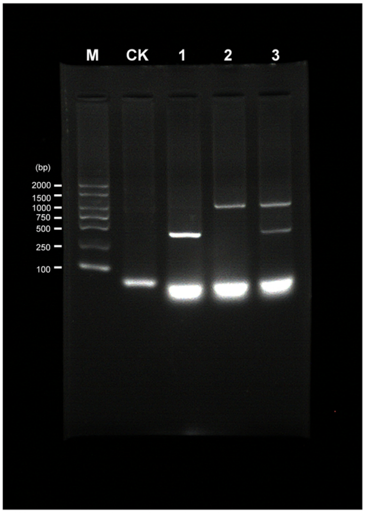

BbTFO1 deletion identification (lanes 1-3) by PCR. Lane M: DNA marker. Lane CK: blank control. Lane 1: WT. Lane 2: Δ*BbTFO1*. Lane 3: Δ*BbTFO1*/*BbTFO1*.
